# Introduction of nirsevimab in Catalonia, Spain: description of the incidence of bronchiolitis and respiratory syncytial virus in the 2023/2024 season

**DOI:** 10.1007/s00431-024-05779-x

**Published:** 2024-09-28

**Authors:** Aida Perramon-Malavez, Víctor López de Rioja, Ermengol Coma, Eduardo Hermosilla, Francesc Fina, Montserrat Martínez-Marcos, Jacobo Mendioroz, Carmen Cabezas, Cristina Montañola-Sales, Clara Prats, Antoni Soriano-Arandes

**Affiliations:** 1 Computational Biology and Complex Systems (BIOCOM-SC) Group, Department of Physics, Universitat Politècnica de Catalunya (UPC), Castelldefels, Barcelona, Catalonia Spain; 2 Primary Care Services Information System (SISAP), Institut Català de La Salut (ICS), Barcelona, Catalonia Spain; 3 Fundació Institut Universitari per a la Recerca a l’Atenció Primària de Salut Jordi Gol i Gurina (IDIAPJGol), Barcelona, Spain; 4 Health Department, Government of Catalonia, Public Health Secretariat, Barcelona, Catalonia Spain; 5 IQS School of Management, Universitat Ramon Llull, Barcelona, Catalonia Spain; 6 Paediatric Infectious Diseases and Immunodeficiencies Unit, Children’s Hospital, Vall d’Hebron Barcelona Hospital Campus, Barcelona, Catalonia Spain

**Keywords:** Nirsevimab, RSV, Respiratory syncytial virus, Bronchiolitis, Epidemiology, Infants

## Abstract

Respiratory syncytial virus (RSV) causes most of the cases of bronchiolitis and thousands of deaths annually, particularly in infants less than 6 months old. In Catalonia (Spain), infants born between April 2023 and March 2024 aged 0–6 months during their first RSV season have been candidates to receive nirsevimab, the novel monoclonal antibody against RSV, since October 2023. We aimed to analyse the dynamics of all-causes bronchiolitis diagnoses and RSV community infections in the current season and compare them to pre-nirsevimab epidemics. We collected epidemiological data from the Information System for Surveillance of Infections in Catalonia (SIVIC) on daily all-causes bronchiolitis clinical diagnoses and RSV-confirmed cases provided by rapid antigen tests in primary care practices. We calculated the rate ratio (RR) for the incidence of all-causes bronchiolitis for children aged 0-11 m-old with respect to 12-35 m-old between September 2014 and January 2024. We analysed the RR of the incidence of RSV-confirmed infection for 0-11 m-old and 12-35 m-old with respect to the > 35 m-old, from January 2021 to January 2024. We then computed the relative difference of the RR, designated as percentage of reduction of risk, between season 2023/2024 and former epidemics. With a global coverage recorded rate for nirsevimab of 82.2% in January 2024, the age-specific 0-11 m-old RR (95% CI) of RSV infection incidence for > 35 m-old was 1.7 (1.5–2.0) in season 2023/2024. The RR (95% CI) had been 7.4 (5.6–9.9), 8.8 (6.9–11.3), and 7.1 (5.7–8.9) in 2020/2021, 2021/2022, and 2022/2023, respectively. Regarding the incidence of all-causes bronchiolitis for the 0-11 m-old group compared to the 12-35 m-old, the pre-pandemic (2014/2015–2019/2020) and 2022/2023 RR (95% CI) were 9.4 (9.2–9.6) and 6.0 (5.7–6.2), respectively, significantly higher than the RR of 3.6 (3.4–3.8) for the most recent season, 2023/2024.

*Conclusion*: Concurring with the introduction of nirsevimab, the risk of RSV infection for infants aged 0-11 m-old compared to > 35 m-old has been reduced by 75.6% (73.4–77.5) in last season, and the risk for all-causes bronchiolitis for 12-35 m-old by 61.9% (60.9–62.9) from the pre-pandemic period and by 39.8% (39.3–40.2) from the 2022/2023 epidemic, despite high RSV community transmission, especially in older infants

**Table:** 

**What is Known:** *• RSV is responsible for approximately 70% of bronchiolitis cases and causes severe disease, particularly in infants < 6 months of age.* *• Nirsevimab effectiveness against RSV-associated disease, particularly hospitalisations, was expected to be around 80%;* *other Spanish regions, such as Galicia and Valencia, and European countries including Luxembourg and Germany, have already reported good results in implementing nirsevimab to prevent RSV-associated hospitalisations and PICU stays.*	
**What is New:** *• We provide insight into the community incidence of RSV and all-causes bronchiolitis for season 2023/2024, when nirsevimab has been introduced to the Catalan population, using.* * primary healthcare data, which enabled us to assess the burden of RSV infections and bronchiolitis in the commonly seasonally saturated primary healthcare practices.* *• Our study reveals that the risk of all-causes bronchiolitis for infants aged 0-11 m-old compared to older infants was reduced by 40% compared to the previous season and 62% compared to pre-pandemic standards, and for RSV infection it was reduced by 76%.*

**Supplementary Information:**

The online version contains supplementary material available at 10.1007/s00431-024-05779-x.

## Introduction

The respiratory syncytial virus (RSV) causes most cases of bronchiolitis (60–80%) and thousands of deaths annually, particularly in infants < 6 months old [1][2]. The RSV epidemic is expected every year between October and March in temperate areas of the northern hemisphere. Although the COVID-19 pandemic altered the circulation of RSV and its seasonal pattern, an epidemic pattern similar to the pre-pandemic was observed again in the 2021/2022 and 2022/2023 seasons, causing severe disease and hospitalisation in infants, especially the youngest.

The European Medicines Agency (EMA) recommended in September 2022 a marketing authorisation for nirsevimab, the novel monoclonal antibody against RSV, to prevent first seasonal episodes of RSV-associated lower respiratory tract disease (LRTI) in infants. Nirsevimab is expected to avoid RSV-associated severe disease by 80% [3]. However, only a few European countries started the campaign for this preventive measure during the 2023/2024 RSV season, and countries such as France [4, 5] and the USA [6] reported difficulties in distributing it due to a limited supply.

In Catalonia (Spain), all infants born between April 2023 and March 2024 (1-year cohort), aged 0 to 6 months during their first RSV season, and high-risk infants during the second season were eligible to receive nirsevimab [7]. This immunisation was broadly administered in primary care practices (PCPs) and public and private hospitals. Coverage is publicly reported [8].

In addition, since the end of 2020, rapid antigen tests (RATs) have been available for paediatricians working in PCP for use in testing for influenza A, influenza B, adenovirus, and RSV. These RATs have been performed on children with respiratory viral infection symptoms. Data on both RSV-confirmed infections with RAT and daily all-causes bronchiolitis clinical diagnoses from patients who attended PHC are publicly available in the Information System for Surveillance of Infections in Catalonia (SIVIC) database [9]. PCP is free and universal in Catalonia.

We aimed to analyse the dynamics of all-causes bronchiolitis diagnoses and RSV-confirmed community infections in the current season once nirsevimab had been introduced, and compare them to previous seasons.

## Materials and methods

### Data acquisition

The open-access SIVIC database was used to extract daily all-causes bronchiolitis clinical diagnoses (September 2014–January 2024) and daily RSV-confirmed cases with RAT (January 2021–January 2024) in paediatric PCP [9]. These electronic medical records are classified by age group (0–11 months old, 12–35 months old, > 35 months old). SIVIC extracts the data from the primary care clinical history (PCCH). All public PCPs in Catalonia use the same PCCH.

We extracted data on aggregated bronchiolitis diagnoses from SIVIC, which by definition include the International Classification of Diseases 10th version (ICD-10) codes J21, J21.0, J21.1, J21.8, and J21.9, although the data cannot be filtered by ICD-10. This disease should be exclusively reported for infants ≤ 24 months, but the data source does not have this limit. Hence, for this analysis, bronchiolitis for infants up to 35 months was considered, taking into account the fact that the greatest burden of the disease affects infants ≤ 24 months.

On another note, the protocol for performing RATs is available in the Supplementary Material. RATs are mostly recommended for symptomatic children ≤ 24 months with viral respiratory infection suspicion but they are not restricted to infants. These tests have been broadly used as a primary diagnostic tool by paediatricians since the end of 2020. The tests used during the study period were JusChek (Tecil), Certest Biotec (Vircell), Flowflex (Alleu), and Ecotest (Linear), depending on availability in the public healthcare system. All of these tests have similar sensitivities and specificities, which are higher when the patient is symptomatic, as was the case in this study, and do not vary with age. Data on RSV-confirmed cases with RAT has been named daily RSV infections throughout the manuscript for simplicity.

Reference populations per year and by age group were also obtained from the SIVIC database. Access to the PCP system is universal and free in Catalonia.

### Statistical analysis

We computed daily incidences of all-causes bronchiolitis disease and RSV infection as reported cases per 100,000 inhabitants. The average pre-pandemic season for description of all-causes bronchiolitis was created as the mean of the epidemics from 2014/2015 to 2019/2020, considering the whole period from September to August. Previous alignment was unnecessary due to the regularity of bronchiolitis epidemic seasonality in Catalonia. The 95% confidence interval (CI) was also provided.

For further analysis, we defined the bronchiolitis or RSV season as the period of 3 months containing the months before, during, and after the epidemic peak, i.e., November to January for all years except 2020/2021, which was delayed to May 2021–July 2021. For 2021/2022 and 2022/2023, it came early, from October to December.

In addition, we comprehensively detailed weekly RSV-infection incidence, RAT incidence which is often referred to as diagnostic effort, and positivity rates—i.e., the percentage of RSV-positive tests in relation to the number of tests performed across the different age groups and study periods. The Mann–Whitney *U* test was employed to compare the distribution of these variables between the most recent season, 2023/2024, and prior seasons 2020/2021, 2021/2022, and 2022/2023, to determine whether this last season has been significantly different to the others. To account for multiplicity, the Bonferroni correction was implemented. We also analysed the percentage of RSV-confirmed infections for each paediatric age group (0-11 m-old, 12-35 m-old, > 35 m-old), computing the percentage of weekly (7-day cumulative) infections corresponding to each age group based on the total number of weekly RSV cases.

In addition, we calculated the incidence rate ratios (RRs) and associated 95% CIs for the incidence of all-causes bronchiolitis in 0-11 m-old from seasons 2014/2015 to 2023/2024. We selected the 12-35 m-old group as a baseline reference. Similarly, the RRs (95% CI) for the incidence of RSV infection in 0-11 m-old and 12-35 m-old from 2021/2022 to 2023/2024 were obtained, with the > 35 m-old group as the baseline reference.

To compare last season, 2023/2024, to pre-nirsevimab epidemics, we calculated the mean pre-nirsevimab RR with its 95% CIs. For bronchiolitis, seasons 2020/2021 and 2021/2022 were excluded, and 2022/2023 was compared separately because of the disruption caused by the COVID-19 pandemic. Moreover, we assessed whether the incidence of bronchiolitis in 2023/2024 was significantly different than in the pre-pandemic period and the 2022/2023 season throughout a Mann–Whitney *U* test, as we did for RSV infections.

The percentage of change in risk for RSV infection or all-causes bronchiolitis was assessed by computing the relative difference of the 2023/2024 RR concerning pre-nirsevimab RRs. All analyses were performed in Python 3.11.9 [10].

The entire code and process, including libraries used and variable names, is available for further reference and replication at https://github.com/BIOCOM-SC/cloud-of-codes/tree/main/Aida_Perramon-Malavez/Nirsevimab.

## Results

### RSV infection description

#### Comparison of 2021/2022 and 2022/2023 RSV epidemics with 2023/2024 by age group

**Fig. 1:**
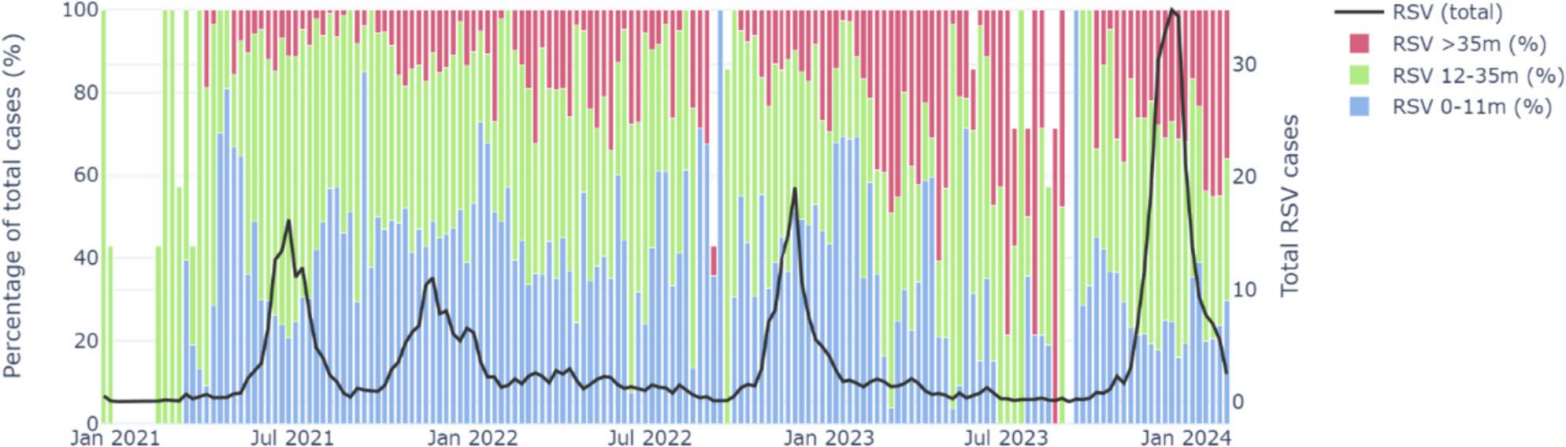
Percentage of weekly RSV infections (left axis) corresponding to 0-11 m-old (blue), 12-35 m-old (green), and > 35 m-old (magenta) concerning total number of RSV infections (black, right axis)

We analysed the percentage of confirmed RSV infections for each age group (Fig. 1). We observe a significant increase in RSV cases in the last season, 2023/2024.

**Fig. 2:**
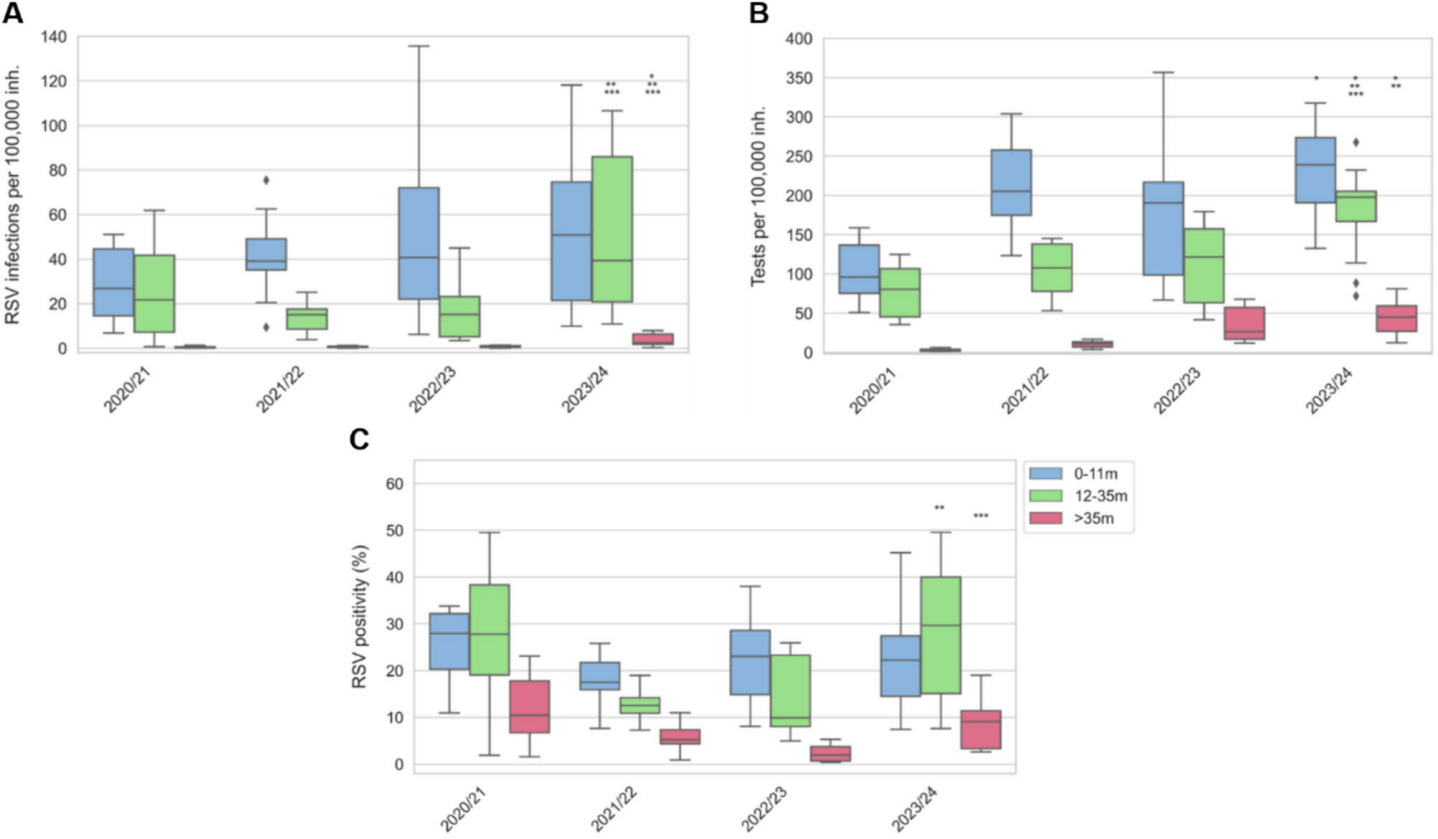
**A** Weekly RSV incidence, **B** diagnostic effort, and **C** positivity for 0-11 m-old (blue), 12-35 m-old (green), and > 35 m-old (magenta) children and the RSV epidemics from 2020/2021 to 2023/2024. The Mann–Whitney *U* test was used to compare medians between seasons 2023/2024 and 2020/2021 (* if *p* < 0.05), 2021/2022 (** if *p* < 0.05), and 2022/2023 (*** if *p* < 0.05). *P*-values were adjusted using the Bonferroni correction

However, positivity rates for all age groups were similar to 2020/2021 (summer 2021) (Fig. 2). During the peak of season 2023/2024, 30% of the RSV infections were in children > 35 m-old, compared to 20% in 0-11 m-olds or 50% in 12-35 m-olds. Hence, regardless of the diagnostic effort, findings suggest a significantly increased community transmission of RSV in children ≥ 12 m-old (*p* < 0.001) when compared to the last two seasons.

#### Associated risk of RSV infection by age, per season

**Fig. 3:**
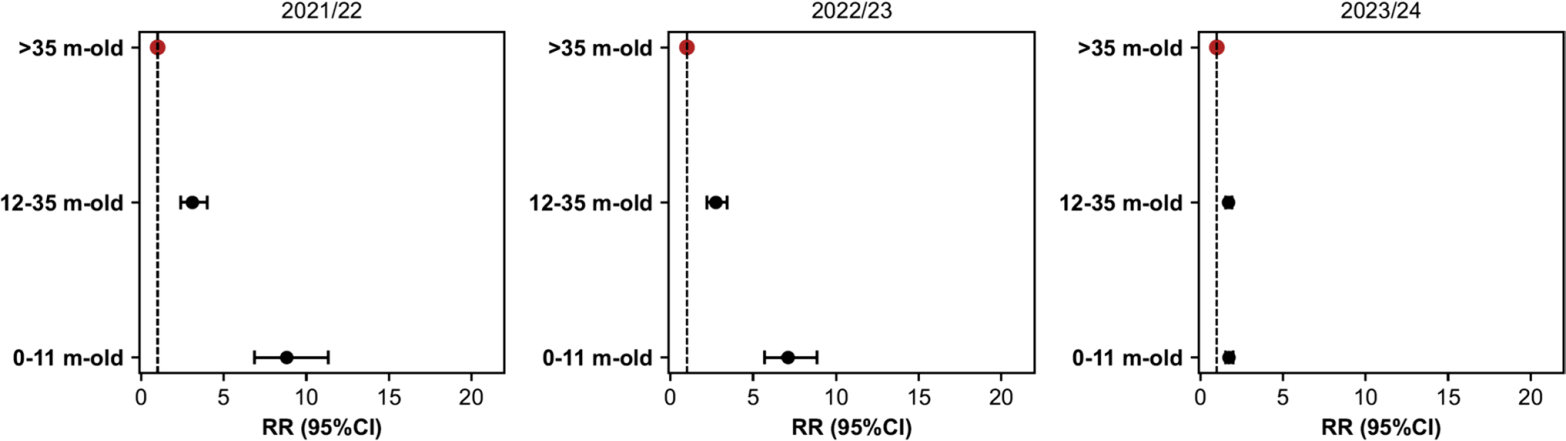
Rate ratio (RR) of RSV infection for age groups 0-11 m-old and 12-35 m-old, compared to > 35 m-olds, for seasons 2021/2022, 2022/2023, and 2023/2024

For the 0-11 m-old group, the RRs (95% CI) before 2023/2024 were 7.4 (5.6–9.9), 8.8 (6.9–11.3), and 7.1 (5.7–8.9) in 2020/2021, 2021/2022, and 2022/2023, significantly higher than the RR (95% CI) of 1.7 (1.5–2.0) in 2023/2024 (Fig. 3). Significant results from the Mann–Whitney *U* test comparing incidences of the periods mentioned above also showed *p* < 0.001.

Hence, in 2023/2024, the risk for RSV infection in infants 0-11 m-old compared to > 35 m-old was reduced by 76.7% (73.0–79.9), 80.3% (78.0–82.5), and 75.6% (73.4–77.5) from 2020/2021, 2021/2022, and 2022/2023 epidemics, respectively.

### All-causes bronchiolitis description

#### Comparison of pre-pandemic and post-pandemic all-causes bronchiolitis epidemics with 2023/2024

**Fig. 4:**
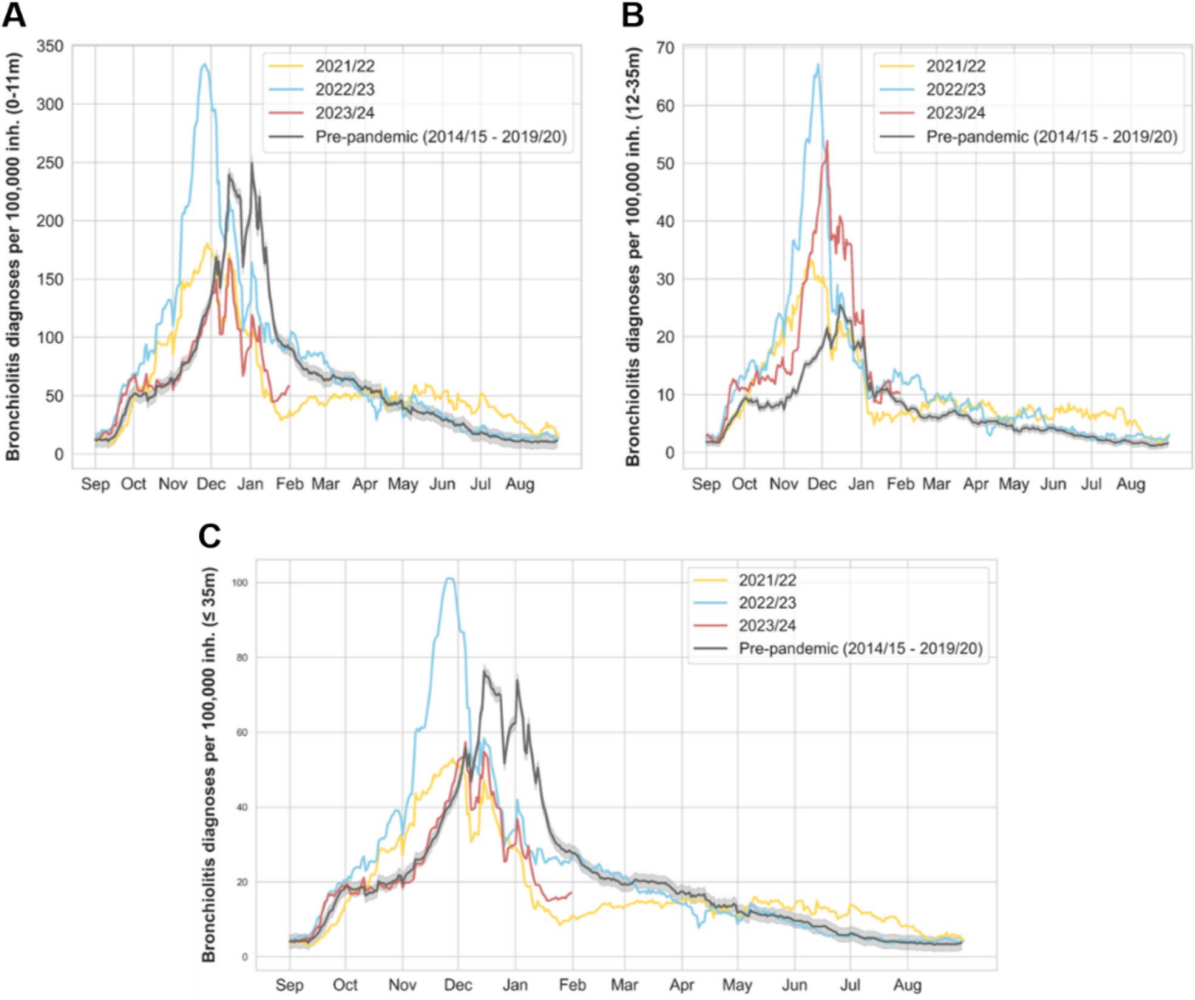
Daily bronchiolitis clinical diagnosis data per 100,000 inhabitants for **A** 0-11 m-old, **B** 12-35 m-old, **C** ≤ 35 m-old. The grey line represents the average pre-pandemic season (2014/2015 to 2019/2020) with 95% CI. The yellow, blue, and red lines correspond to seasons 2021/2022, 2022/2023, and 2023/2024, respectively

We depicted the incidence of all-causes bronchiolitis across infants 0-11 m-old, 12-35 m-old, and the combined group (all of them, ≤ 35 months), comparing the average incidence from pre-pandemic seasons (95% CI) with the last three epidemics (Fig. 4). In the 2023/2024 season, bronchiolitis incidence notably decreased compared to pre-pandemic years among 0-11 m-olds. Conversely, incidence increased among 12-35 m-old children, in line with the ongoing post-pandemic trend. Analysis of the epidemic’s overall impact on the combined ≤ 35 m-old age group revealed a pattern consistent with that observed in the 0-11 m-olds, as expected due to the higher disease burden in this cohort. This analysis indicated an overall reduction in bronchiolitis incidence during the 2023/2024 season, with seasonality closely resembling the pre-pandemic average.

#### Associated risk of all-causes bronchiolitis by age and by season

For the 0-11 m-old group compared to the 12-35 m-old, the pre-pandemic and 2022/2023 RRs (95% CI) were 9.4 (9.2–9.6) and 6.0 (5.7–6.2), respectively, significantly higher than the RR of 3.6 (3.4–3.8) for 2023/2024. Significance results from the Mann–Whitney *U* test comparing incidences of the aforementioned periods also showed *p* < 0.001 (Supplementary Material).

Hence, in 2023/2024, the risk for all-causes bronchiolitis in infants 0-11 m-old compared to 12-35 m-old was reduced by 61.9% (60.9–62.9) from the pre-pandemic period and by 39.8% (39.3–40.2) from the 2022/2023 epidemic.

## Discussion

Since October 2023, nirsevimab has been recommended in Catalonia, financed with public funds, and administered in all PCPs and hospitals as a preventive measure to protect all children against RSV in their first season and high-risk children in their second RSV season. It was expected to reduce severe disease by 80% [11]. Our findings suggest that the risk of RSV infection for infants aged 0-11 m-old compared to > 35 m-olds has been reduced by 75.6% (73.4–77.5) since last season, and the risk for all-causes bronchiolitis compared to 12-35 m-olds by 61.9% (60.9–62.9) from the pre-pandemic period, and by 39.8% (39.3–40.2) from the 2022/2023 epidemic, despite a high RSV community transmission, especially among children aged ≥ 12 months. Remarkably, the reduction in RR for bronchiolitis diagnoses would be more significant if RSV-bronchiolitis were considered alone. However, these specific data are not publicly available at the primary care level, only aggregated data for all-causes bronchiolitis is accessible.

On the other hand, the significant increase in RSV cases in 2023/2024 could be attributed to increased diagnostic efforts for age groups ≥ 12 months. However, positivity rates in all age groups were similar to those observed in 2020/2021 (summer 2021), the first epidemic wave of RSV during the COVID-19 pandemic. During the first year of the pandemic, RSV circulation was very low and consequently a larger cohort of individuals had not been previously exposed to RSV and were therefore more susceptible to infection. This phenomenon, known as immunity debt, explains the higher proportion of RSV infections in the 12–35 month age group during that season [12]. When comparing positivities within the same age range, we are relativizing by the number of tests performed and therefore we can infer that this 2023/2024 season a proportion of ≥ 12 m-age was infected with RSV similar to that of the 2020/2021 season now without the hypothesis of immunity debt but with an explanation of greater community transmission.

The coverage rate for nirsevimab for those born between April and September 2023 has been 88%, and the global coverage rate for children under 1 year of age, estimated until the end of January 2024, is 82.2% [8]. Note that there is a delay in data recording; therefore, these percentages might be even higher than reported.

Unlike other previously published studies about reducing RSV-associated hospitalisation with nirsevimab [13, 14], our study used PCP data, which contains the greatest healthcare burden as it is primary healthcare and because it is universal and free, hence with a larger sample size and study period from September 1, 2014, to January 31, 2024. Additionally, we provided the RR of incidence of RSV infection and all-causes bronchiolitis diagnosis of infants eligible for nirsevimab compared to non-eligible cohorts, allowing for quantifying the consequences of the immunisation on the RSV epidemics and potentially related severe disease (bronchiolitis).

Similarly, concerning the last epidemic period, we found a substantial reduction in all-causes bronchiolitis for 0-11 m-old. Additionally, there were fewer cases of RSV infection in this age group. Nonetheless, we observed more all-causes bronchiolitis cases and a higher incidence of RSV infection in older infants, particularly 12-35 m-olds, pointing to a high-circulation epidemiological context in the absence of immunisation. Previously published studies mentioned an increased mean age of hospital-admitted children but did not provide a comprehensive age-specific analysis [13, 14].

Comparable findings have been observed in Galicia, another autonomous community of Spain that introduced nirsevimab in the autumn of 2023 [15, 16]. In this region, there was also noted to be increased community transmission of RSV, although lower incidence levels of bronchiolitis and RSV infections in 0-11 m-olds.

Interestingly, RSV positivity for 0-4y-old infants in Germany in 2023/2024 was similar to the previous year [17], even after the implementation of immunisation. After introducing immunisation in France, bronchiolitis in children < 35 m-old reached pre-pandemic incidence levels during season 2023/2024, slightly lower than in 2022/2023 [18]. These differences may be attributed to the divergent immunisation protocols and coverage rates and the reported irregularities in nirsevimab supply [5].

As for limitations, we must consider that compliance with the protocol for testing children with a RAT is not necessarily homogeneous among PCP paediatricians despite its availability and that are publicly financed; therefore, data about community incidence cannot be deduced from RSV-confirmed cases. Nevertheless, we assume there have not been significant changes over time in each particular PCP, so season-to-season compatibility is still feasible. Also, aggregated epidemiological data do not include relevant confounders like those related to socioeconomic factors, which could bias the results. Nonetheless, the magnitude of the measured impact is consistent with other results, thus pointing to a minor effect of these confounders. Moreover, when calculating the RR, a historically consistent baseline group should be used to provide robustness when comparing periods. For this reason, the > 35 m-old was used as the control group to assess the divergence in the incidence of RSV infections among epidemics since, in each epidemic, the number of infected > 35 m-olds was similar. However, for all-causes bronchiolitis, we were limited by the diagnosis criteria and the age resolution of the reported data. Thus, we used 12-35 m-olds as the control group, which may have introduced bias into our results. Additionally, recommended immunization was implemented for infants under 6 months old, so the reduction might be underestimated since the main outcome age range includes children up to 11 months old. Not being able to disaggregate the data by age is a limitation that cannot be compensated for, but that does not significantly impact our conclusions since children born from April 2023 were immunized and the data was extracted in January 2024. Nonetheless, this limitation would only result in an underestimation of the effect of nirsevimab. In fact, the reductions in RR that we have assessed cannot be quantitatively compared with the outcomes of clinical studies, such as effectiveness, due to differences in objective and study design (e.g. use of RAT instead of PCR, or a population-level focus rather than a patient-level one). In this sense, our study is centred on a real-world evaluation and, as such, relies on the standard practices of paediatricians in primary care settings in Catalonia.

Despite this, our study has several strengths. Using primary care data, we account for all children attending PCP in Catalonia, potentially 1 million children [19] since healthcare is universal and free. Moreover, while most previous studies only reviewed hospitalisation data, we have provided a depiction of the changes in primary healthcare outcomes since the introduction of nirsevimab, which we have not found in any previous report. Therefore, this study provides epidemiological context for this 2023/2024 RSV season in Catalonia, which can be used for other related studies attempting to estimate the effectiveness of nirsevimab, such as the Coma et al. [20] study that assesses the effectiveness of nirsevimab in hospital and primary healthcare outcomes in Catalonia. Finally, the quality and completeness of the SIVIC database are to be noted, as it is used for surveillance of respiratory infections in Catalonia and has served in numerous studies [21–23]                                                                                                                                                                                                                                                                                                                                                                                                                                                                                                                                                                    .

## Conclusion

This 2023/2024 season showed a significantly lower comparative risk of RSV infection incidence (by 75.6% (73.4–77.5)) and all-causes bronchiolitis incidence (by 39.8% (39.3–40.2)) than the previous season, in infants 0-11 m-old, even in a high RSV community burden season in Catalonia. 12-35 m-old children carried the main burden of community RSV infection in season 2023/2024. During the season, there were significantly greater numbers of incidence of all-causes bronchiolitis and RSV infection in children ≥ 12 m-old. These differences from previous seasons may be attributed to the introduction of the immunisation campaign with nirsevimab.

## Supplementary Information

Below is the link to the electronic supplementary material.

Supplementary file1 (PDF 830 KB)

## Data Availability

Data is provided within the manuscript or supplementary information files.
