# Supplementary material for: Introduction of nirsevimab in Catalonia, Spain: description of the incidence of bronchiolitis and respiratory syncytial virus in the 2023/2024 season
Source: Eur J Pediatr. 2024 Sep 28;183(12):5181–9. doi: 10.1007/s00431-024-05779-x (PMC11527896; doi:10.1007/s00431-024-05779-x)
Supplement: Supplementary file 1 — Supplementary file1 (PDF 830 KB) [file 431_2024_5779_MOESM1_ESM.pdf]

Supplementary Material for: Introduction of nirsevimab in Catalonia, Spain: description of bronchiolitis and the Respiratory Syncytial Virus incidence in the 2023/24 season.

Protocol for RAT performance (Catalan version only):

Tests ràpids d'infeccions respiratòries pediàtriques

Desembre 2020

/Salut Generalitat de Catalunya

SOCIETAT CATALANA DE PEDIATRIA

Índex

1. Justificació 3
2. Protocol de diagnòstic ràpid a pediatria d'atenció primària en època de COVID-19 4
3. Protocol de diagnòstic a pediatria d'atenció primària de COVID-19 5
Annex 1. JUSCHECK (TECIL) 6
Annex 2. CERTEST (VIRCELL) 7

Autors

Laia Asso — Àrea Maternoinfantil de la Secretaria de Salut Pública

Manel Enrubia — Director del Centre Atenció Pediàtrica Integral Barcelona Esquerra (ICS)

Maria Esteller — Equip d'Atenció Primària Tortosa Est i Tortosa Oest (SCP)

Josep de la Flor — CAP El Serral. Sant Vicenç dels Horts (ICS)

Juanjo Garcia — Cap del Servei de Pediatria. Hospital Sant Joan de Déu (SCP)

Anna M. Gatell — Equip de Pediatria Territorial Alt Penedès-Garraf (SCP)

Carles Luaces — Cap del Servei d'Urgències. Hospital Sant Joan de Déu (SCP)

Vicente Morales — Equip de Pediatria Territorial Alt Penedès-Garraf (SCP)

Valentí Pineda — President de la Societat Catalana de Pediatria (SCP)

Maria J Pueyo — Gerència de Processos Integrats. Àrea Assistencial (CatSalut)

Carlos Rodrigo Gonzalo de Liria — Director Clínic de Pediatria. Gerència Territorial Metropolitana Nord. Hospital Germans Trias i Pujol (ICS)

Antoni Salvà — Director Assistencial de l'Equip de Pediatria CAPSBE. Consorci d'Atenció Primària de Salut Barcelona Esquerra (CSC)

Pere Soler — Cap de la Unitat de Malalties Infeccioses i Immunodeficiències Pediàtriques. Hospital Vall de Hebron (SCP)

Marta Urgellès — Coordinadora de Pediatria. Mútua de Terrassa (UCH)

1. Justificació

- L'atenció als infants suposa al voltant de 7,5 milions de visites anuals a l'atenció primària. D'aquestes, al voltant d'1,6 milions estan relacionades amb les infeccions respiratòries. Durant el període epidèmic hivernal l'activitat d'atenció primària s'incrementa un 66% respecte al període no epidèmic, amb un increment de 16.000 visites setmanals per infeccions respiratòries i síndromes gripals.

- La majoria de les infeccions són víriques i les més freqüents estan originades per rinovirus, adenovirus, parainfluença, influença i virus respiratori sincític. Cada any es produeixen al voltant de 3.500 ingressos per infeccions respiratòries víriques i síndromes gripals.

- El diagnòstic diferencial entre les infeccions respiratòries pediàtriques és difícil en infants, una dificultat que està en relació inversa amb l'edat. La possibilitat de fer un diagnòstic etiològic precís permet:
    - disminuir la taxa de reconsultes espontànies, sovint associades a un procés febril prolongat i inespecífic;
    - reduir la utilització d'exploracions complementàries injustificades i poc rendibles;
    - disminuir l'ús inapropiat d'antibiòtics al voltant del 20-25%;
    - incrementar la prescripció d'antivirals en pacients de risc.

- La pandèmia obliga a descartar la infecció per COVID-19 en els infants que es presenten amb símptomes d'infecció respiratòria. La incertesa del diagnòstic, donada la clínica i la sensibilitat dels tests disponibles, i el diferent tractament que s'ha de fer en el cas d'una infecció per SARS-CoV-2, fa necessari posar a disposició dels professionals d'atenció primària totes les eines disponibles per poder millorar el diagnòstic diferencial de les infeccions respiratòries. Per aquesta raó, s'ha considerat necessari dotar les consultes de pediatria d'atenció primària de tests ràpids d'infeccions respiratòries (VRS, influença A i B i adenovirus).

- El protocol que es presenta ha estat elaborat per un conjunt de pediatres que representen la Societat Catalana de Pediatria, les entitats proveïdores agrupades en les patronals (UCH i CSC) i l'Institut Català de la Salut. És un protocol clínic però que té en consideració la situació epidemiològica actual de pandèmia per COVID-19. Com qualsevol protocol d'aquest període 2020-21, haurà d'anar canviant conforme es disposi de més coneixement sobre la COVID-19.

- Els resultats dels tests ràpids han de ser interpretats sempre en el context de la clínica que presenta el pacient.

2. Protocol de diagnòstic ràpid a pediatria d'atenció primària en època de COVID-19

PROVA	INDICACIONS	ESPECIFICACIONS
**Test antigènic ràpid per a grip, VRS i adenovirus**	**Bronquiolitis**	Primer episodi d'infecció respiratòria (febre, rinorrea, tos), que associa a l'auscultació crepitants i/o sibilàncies, en infant < 2 anys,<br>en absència d'altres causes que ho puguin provocar,<br>només en temporada d'epidèmia de VRS.
	**Febre sense focus**	Febre ≥ 38ºC de > 24 hores d'evolució,<br>en infant 3 mesos – 2 anys, prèviament sa,<br>amb triangle d'avaluació pediàtrica estable,<br>amb exploració física normal,<br>només en temporada d'epidèmia gripal.
	**Síndrome gripal**[1]	Febre ≥ 38ºC,<br>*associada a algun dels símptomes següents:*<br>miàlgies, cefalea, malestar general, símptomes gastrointestinals (només en < 3 anys), tos, rinorrea i/o odinofàgia,<br>i *que compleixi algun dels dos criteris següents*:<br>a) < 2 anys amb febre ≥ 38ºC de > 48 hores d'evolució associada a algun dels símptomes esmentats i/o<br>b) de qualsevol edat amb algun factor de risc de quadre greu i segons criteri mèdic,<br>només en temporada d'epidèmia gripal.
**Test d'estreptococ A**	**Faringoamigdalitis aguda (FAA) de probable etiologia estreptocòccica**	- ≥ 3 anys amb clínica de FAA, en absència de símptomes suggestius d'infecció vírica: rinitis, estridor, vesícules, úlceres al paladar, etc.<br>- < 3 anys amb clínica de FAA, i contacte estret amb pacients amb FAA per estreptococ β-hemolític del grup A confirmat o amb signes molt predictius d'etiologia estreptocòccica, com exantema escarlatiniforme o clínica d'estreptococs.

1 En > 6 anys amb quadre clínic compatible, en temporada d'epidèmia gripal i que no presenten criteris per realitzar el **test antigènic ràpid per grip, VRS i adenovirus,** es recomana establir un diagnòstic clínic de **grip** ja que les manifestacions són habitualment característiques.

3. Protocol de diagnòstic a pediatria d'atenció primària de COVID-19

PROVA	INDICACIONS	ESPECIFICACIONS
**Test antigènic ràpid i/o PCR per SARS-CoV-2** segons el protocol vigent[2]	**Cas sospitós d'infecció per SARS-CoV- 2**[2]	Qualsevol persona amb un quadre clínic d'infecció respiratòria aguda d'aparició sobtada de qualsevol gravetat que cursa, entre d'altres, amb febre, tos o sensació de manca d'aire. Altres símptomes com l'odinofàgia, anòsmia, agèusia, dolors musculars, diarrea, dolor toràcic o cefalea, entre d'altres, poden ser considerats també símptomes de sospita d'infecció per SARS-CoV-2 segons criteri clínic.[2]

2 El protocol vigent és el *Procediment d'actuació enfront de casos d'infecció pel nou coronavirus SARS-CoV-2. Actualitzat: 18.10.2020.* https://canalsalut.gencat.cat/web/.content/_A-Z/C/coronavirus-2019-ncov/material-divulgatiu/procediment-actuacio-coronavirus.pdf.

Annex 1. JUSCHECK (TECIL)

Adenovirus / RSV / Influença A+B Combo Rapid Test Cassette (hisop nasofaringi)

Test ràpid d'immunocromatografia per a la detecció d'antígens d'adenovirus, VRS i influença A i B sobre mostra nasofaríngia.

Instruccions

1. Dipositar 15 gotes de la solució reactiva en el tub d'extracció.
2. Introduir-hi l'hisop i donar-li voltes durant 10 segons mentre es prem el tub contra l'hisop.
3. Treure'l esprement el màxim possible el líquid de l'hisop.
4. Posar el comptagotes a l'extrem.
5. Posar la placa sobre una superfície plana i dipositar 3 gotes a cada mostra.
6. Llegir-ne els resultats als 15 minuts (més enllà dels 20 minuts la lectura no és vàlida).

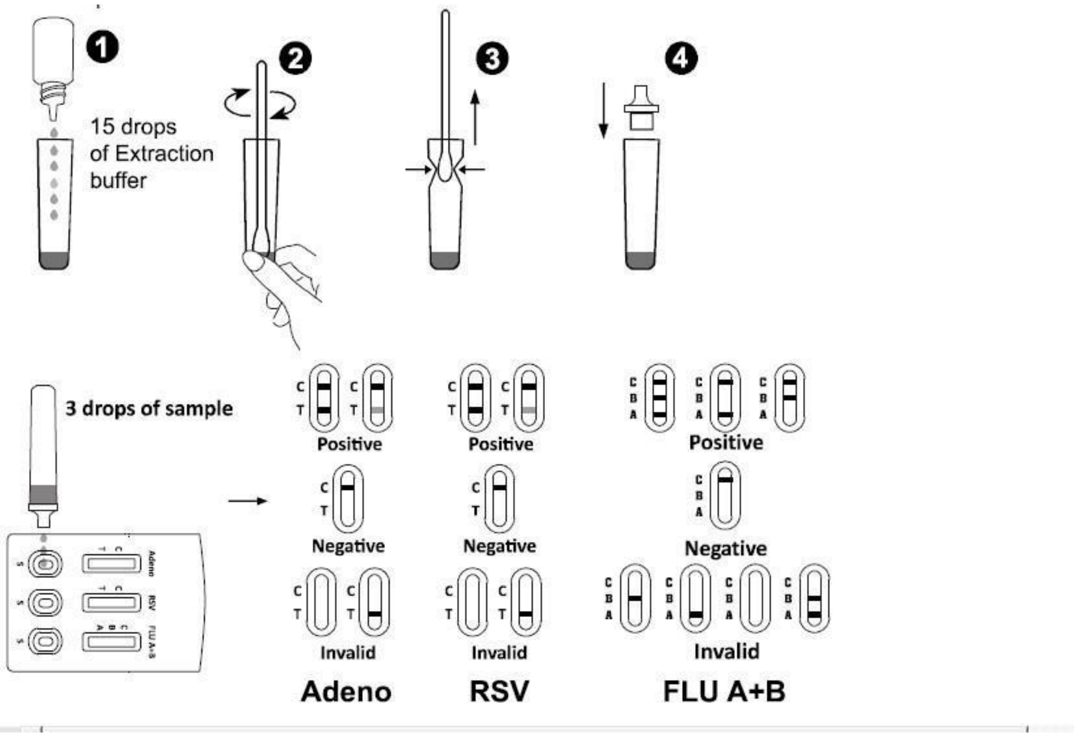

Annex 2. CERTEST (VIRCELL)

CERTEST *Influença A + B + RSV + adeno resp*.

Test ràpid d'immunocromatografia per a la detecció d'antígens d'adenovirus, VRS i influença A i B sobre mostra nasofaríngia.

Instruccions

1. Introduir-hi l'hisop i donar-li voltes durant 1 minut mentre es prem el tub contra l'hisop.
2. Treure'l esprement el màxim possible el líquid de l'hisop.
3. Agitar el tub durant 1 minut.
4. Tallar la punta del tap.
5. Posar la placa sobre una superfície plana i dipositar 4 gotes a cada una de les 4 finestres (A-D).
6. Llegir-ne els resultats als 10 minuts (més enllà dels 10 minuts la lectura no és vàlida).

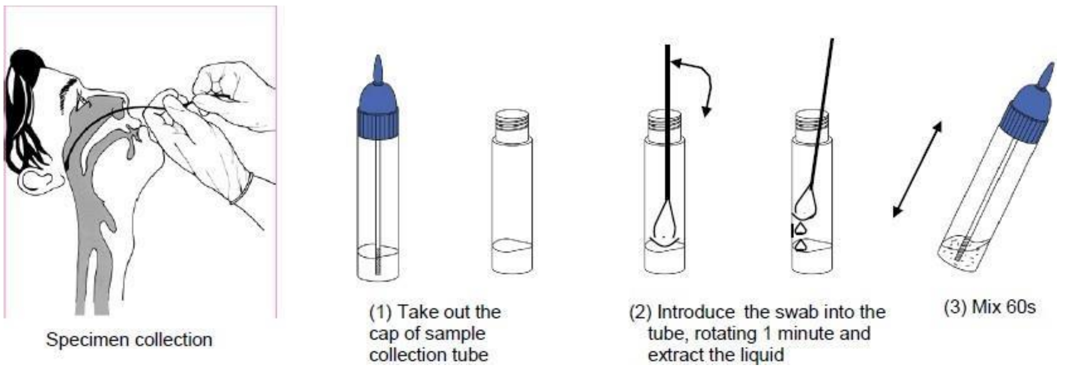

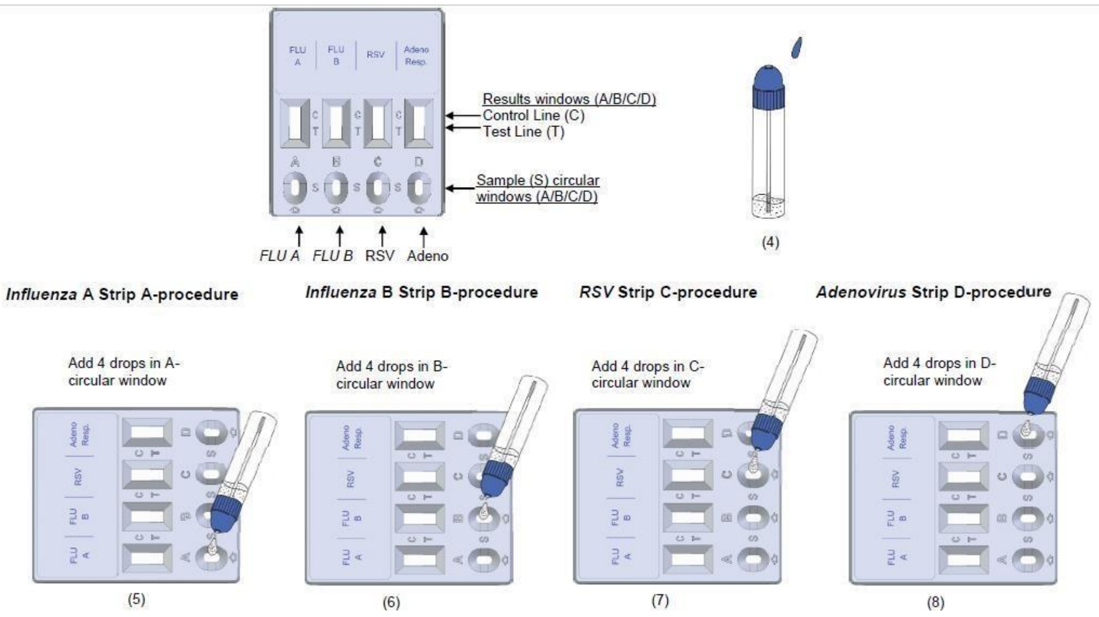

Interpretació dels resultats

Línia vermella resultat positiu
Línia verda control

VALID RESULTS

1.

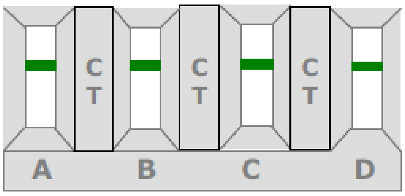

A: Green→Negative *FLU A*
B: Green→Negative *FLU B*
C: Green→Negative *RSV*
D: Green→Negative *Adeno*

15.

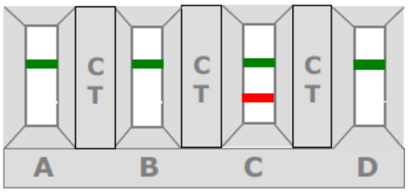

A: Green→Negative *FLU A*
B: Green→Negative *FLU B*
C: Green/Red→Positive *RSV*
D: Green→Negative *Adeno*

13.

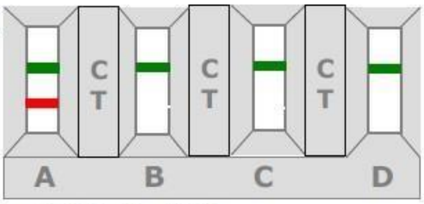

A: Green/Red→Positive *FLU A*
B: Green→Negative *FLU B*
C: Green→Negative *RSV*
D: Green→Negative *Adeno*

14.

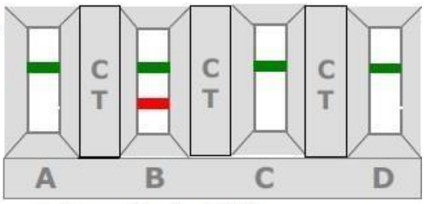

A: Green→Negative *FLU A*
B: Green→Positive *FLU B*
C: Green/Red→Negative *RSV*
D: Green→Negative *Adeno*

16.

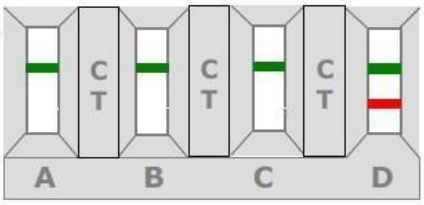

A: Green→Negative *FLU A*
B: Green→Negative *FLU B*
C: Green→Negative *RSV*
D: Green/Red→Positive *Adeno*

RRs for all-causes bronchiolitis:

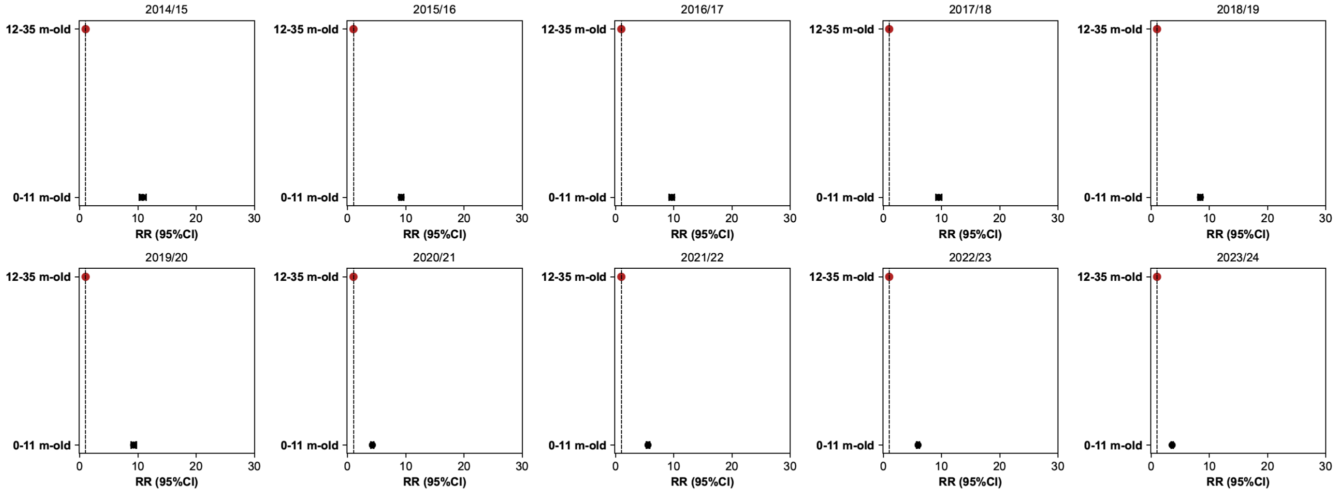

Tables of cases and population considered per season for RSV:

Season	RSV 0-11 m-old	RSV 12-35 m-old	RSV >35 m-old	Population 0-11 m-old	Population 12-35 m-old	Population >35 m-old
2020/21	194.71	423.86	62	55543	131380	956610
2021/22	274.71	242.14	77.86	50007	125037	955363
2022/23	314.57	285.57	103.57	50538	118319	944280
2023/24	361.14	805.86	472.43	51443	116596	940813

Tables of cases and population considered per season for all-causes Bronchiolitis:

Season	Bronchiolitis 0-11 m-old	Bronchiolitis 12-35 m-old	Population 0-11 m-old	Population 12-35 m-old
2014/15	7459.43	1691.29	64267	157338
2015/16	9063.71	2265.43	63979	146969
2016/17	8432.86	2004.71	61589	141503
2017/18	7536.43	1827.29	61671	142124
2018/19	8100.29	2256	59322	139559
2019/20	7684.57	2005.57	57027	137446
2020/21	2827.86	1576.57	55543	131380
2021/22	5408.29	2423.43	50007	125037
2022/23	8226.14	3232.29	50538	118319
2023/24	4338	2739.43	51443	116596
Pre-pandemic	48277.29	12050.29	367855	864939
